# Peculiar liquid-feeding and pathogen transmission behavior of *Aedes togoi* and comparison with *Anopheles sinensis*

**DOI:** 10.1038/srep20464

**Published:** 2016-02-03

**Authors:** Sang Joon Lee, Dooho Kang, Seung Chul Lee, Young-Ran Ha

**Affiliations:** 1 School of Interdisciplinary Biosciences and Bioengineering, Pohang University of Science and Technology, Pohang, 790-784, Republic of Korea; 2 Center for Biofluid and Biomimic Research, Pohang University of Science and Technology, Pohang, 790-784, Republic of Korea; 3 Department of Mechanical Engineering, Pohang University of Science and Technology, Pohang, 790-784, Republic of Korea; 4 Division of Integrative Bioscience and Bioengineering, Pohang University of Science and Technology, Pohang, 790-784, Republic of Korea

## Abstract

Female mosquitoes transmit various diseases as vectors during liquid-feeding. Identifying the determinants of vector efficiency is a major scientific challenge in establishing strategies against these diseases. Infection rate and transmission efficiency are interconnected with the mosquito-induced liquid-feeding flow as main indexes of vector efficiency. However, the relationship between liquid-feeding characteristics and pathogen remains poorly understood. The liquid-feeding behavior of *Aedes togoi* and *Anopheles sinensis* was comparatively investigated in conjunction with vector efficiency via micro-particle image velocimetry. The flow rates and ratio of the ejection volume of *Aedes togoi* were markedly higher than those of *Anophels sinensis*. These differences would influence pathogen re-ingestion. Wall shear stresses of these mosquito species were also clearly discriminatory affecting the infective rates of vector-borne diseases. The variations in volume of two pump chambers and diameter of proboscis of these mosquito species were compared to determine the differences in the liquid-feeding process. Liquid-feeding characteristics influence vector efficiency; hence, this study can elucidate the vector efficiency of mosquitoes and the vector-pathogen interactions and contribute to the development of strategies against vector-borne diseases.

Mosquitoes (Diptera: Culicidae) are medically important insects causing vector-borne disease, such as malaria and Japanese encephalitis. Strategies against these diseases have been established by mosquito control, vaccination, and medicines. Biological problems such as development of resistance to drugs and insecticides remain a huge burden despite strategies and massive research efforts^1^. To address these problems, attempts have been made to reveal the interactions of mosquitoes with hosts and pathogens. The key to this research area is the determination of the liquid-feeding behaviors of female mosquitoes and the correlated trade-offs in pathogen-mosquito interactions. Mosquitoes attempt to increase reproduction, and pathogens enhance their transmission^2^. Detailed understanding of these interactions can elucidate the mechanism of pathogen transmission and provide a novel perspective for disease control^3^.

Vector-pathogen interactions contribute to the overall dynamics of infectious diseases. The vector efficiency of mosquitoes has been widely investigated to disrupt the transmission of mosquito-borne diseases^4^. Three main aspects are evaluated: the infection rate of the vector (pathogen-positive in the abdomen), transmission rate (pathogen-positive in saliva), and transmission efficiency (pathogen-positive in the host)^4^,^5^,^6^. Vector efficiency among mosquito species differs considerably under various environmental conditions^4^. A major scientific issue is what determines the vector efficiency of mosquitoes^4^.

The vector efficiency of a mosquito species may be closely interconnected with its intrinsic liquid-feeding behavior^7^. Pathogens manipulate the liquid-feeding behavior of mosquitoes to enhance the transmission efficiency^8^. Pathogens can interfere in the intake rate^7^, probing behavior^9^, feeding persistence^10^,^11^, and locomotor activity^12^,^13^ of female mosquitoes during the liquid-feeding. Mosquitoes transmit pathogens during the liquid-feeding process; thus, the target behavior is closely associated with liquid-feeding flow^14^. The systaltic movements of the salivary pump and the two feeding pump chambers regulate the liquid flow during feeding^15^. They also directly influence the efficiency of pathogen delivery^16^,^17^. Therefore, the liquid-sucking ability of the pump organs of a female mosquito can significantly determine vector efficiency.

Few studies have been conducted on the liquid-feeding flow of a single mosquito species^18^,^19^,^20^,^21^. In addition, these studies mainly focused on the efficient pumping system of mosquitoes and not on pathogen delivery. The relationship between the liquid-feeding behavior and pathogen transmission remains poorly understood. Moreover, various mosquitoes with similar morphologies can exhibit distinctly different liquid-feeding behaviors because of dissimilar mechanical characteristics^22^. Therefore, various mosquito species can present different liquid-feeding flow characteristics closely associated with vector efficiency.

In the current study, we compared the liquid-feeding behavior of *Aedes togoi* and *Anopheles sinensis* which exhibit different vector efficiencies. *Aedes togoi* is manly involved in the transmission of a wide species of filariae^23^, yellow fever^24^, and Japanese encephalitis (JE)^25^ in Southeast Asia and the Pacific coast of Canada and USA^26^. *Anopheles sinensis* has been known to transmit human malaria (P. vivax), filariasis, and JE virus, especially in the Oriental region and the contiguous parts of the eastern Palaearctic region^27^. The liquid-feeding processes of the two species were measured by micro-particle image velocimetry (PIV) to distinguish their vector efficiencies based on their liquid-feeding flow characteristics. Additionally, the morphological factors and systaltic movements of the two pump organs of two mosquito species were evaluated by synchrotron X-ray microscopic computed tomography (SR-μCT). These results were comparatively analyzed with respect to the liquid-feeding flow characteristics. The experimental results can elucidate the intrinsic liquid-feeding characteristics of the mosquitoes and their effects on vector efficiency.

## Results

### Liquid-feeding flow characteristics

**Figure 1:**
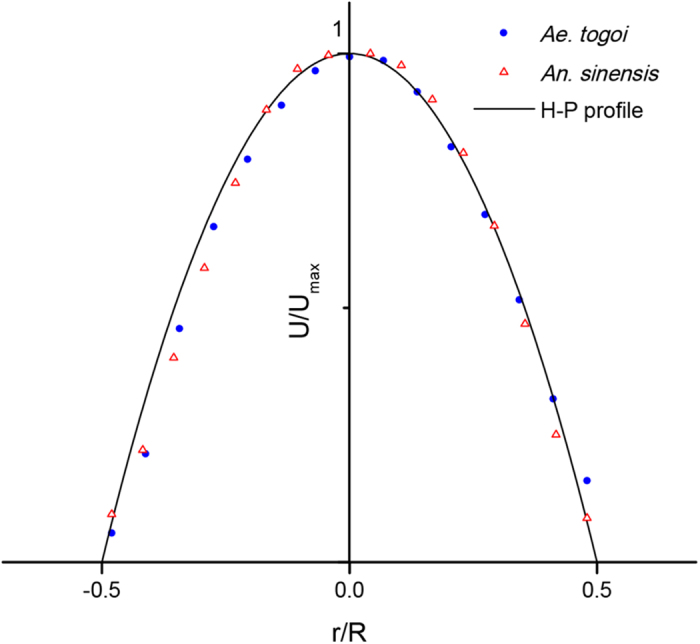
Parabolic velocity profiles of the liquid-feeding flow inside the food canal of *An. sinensis* and *Ae. togoi*. Velocity profiles of the two mosquito species were measured by micro-PIV. R is the inner radius of the food canal in the proboscis and r is the radial distance from the center of the food canal. The velocity profiles inside the food canal exhibit a parabolic profile of the Hagen-Poiseuille flows (H-P profile). The Reynolds and the Strouhal number satisfy the conditions of the Hagen-Poiseuille flow (Re: 0.61 ± 0.29 and 0.28 ± 0.05, St: 0.008 ± 0.002 and 0.021 ± 0.004 for *Ae. togoi* and *An. sinensis*, respectively).

To comparatively evaluate the liquid-feeding abilities of the two female mosquito species, the velocity field information of liquid flow inside their food canals were measured by micro-PIV. The velocity fields show pulsatile phasic variations caused by the systaltic motion of the cibarial pump and the pharyngeal pump^18^. The Reynolds number (Re) and the Strouhal number (St) inside the food canal presented small values; thus, the liquid-feeding flow rates of *Ae. togoi* and *An. sinensis* can be evaluated under the assumption of the Hagen-Poiseuille flow^14^,^18^. The velocity profiles for both mosquito species, depicted in Fig. 1 are in good agreement with the parabolic velocity profile of the Hagen-Poiseuille flows. For a Newtonian fluid, the flow rate (Q) is estimated as follows:


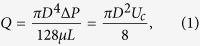


where Δ*P* is the differential pressure generated by the pump muscles, *D* and *L* are the diameter and length of the food canal, *μ* is the viscosity of the working fluid, and *U*_*c*_ is the velocity at the center of the food canal.

**Figure 2:**
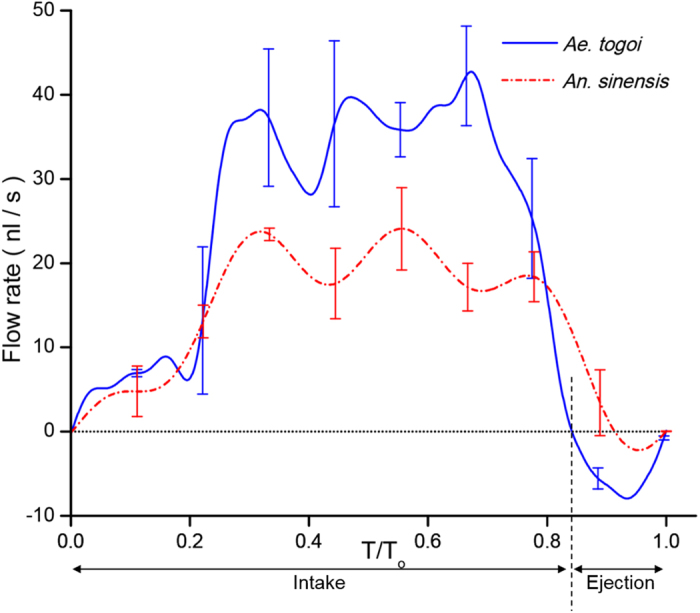
Phasic variations in flow rates inside the food canal of *An. sinensis* and *Ae. togoi*. Temporal variations of flow rates were evaluated under the assumption of Hagen-Poiseuille flow. Liquid-feeding process is divided into two stages: the intake stage and the ejection stage. All data are expressed as mean ± s. d. T/T_o_ represents the dimensionless time normalized by the liquid-feeding period T_o_. Error bars represent the standard deviation.

**Figure 3:**
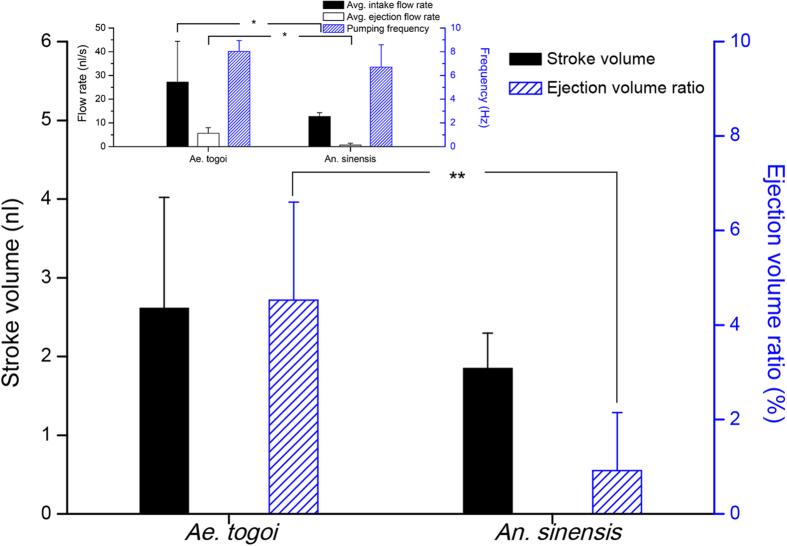
Comparison of the stroke volume and ejection volume ratio between *Ae. togoi* and *An. sinensis*. The stroke volume was obtained by dividing the average flow rate by the pumping frequency. The average flow rates in the intake and the ejection stages, as well as the pumping frequency, were obtained from the flow rates of the two mosquito species. The results are shown in inset. Error bars represent the standard deviation; *P < 0.05, **P < 0.01.

The flow rates of *Ae. togoi* and *An. sinensis* were evaluated using Eq. (1) to compare their liquid-feeding abilities. The overall flow rates and liquid-feeding flow patterns of the two mosquito species were compared. Figure 2 shows the typical variations of phase-averaged flow rates in *Ae. togoi* and *An. sinensis*. The time scales for both mosquitoes were normalized by the corresponding feeding period. Their pumping frequencies are similar, as shown in Fig. 3. The general patterns of the liquid-feeding processes of *Ae. togoi* and *An. sinensis* are comparable and composed of two distinct stages, that is, the intake and the ejection stages. However, the overall flow rates in the two stages are quite different in *Ae. togoi* and *An. sinensis*. The proportions of the ejection stage to the feeding period are also different (15.3 ± 6.7% and 5.6 ± 5.1% for *Ae. togoi* and *An. sinensis*, respectively, P < 0.05).

The intake volume in one feeding period (stroke volume) and the ratio of ejection volume to the stroke volume were quantitatively evaluated to contrast the flow rate and the ratio of the ejection stage (Fig. 3). The stroke volume was derived by dividing the average flow rate by the pumping frequency, the number of occurrences of repeated feeding cycle per one second. The average flow rate of *Ae. togoi* during the intake stage is 27.3 ± 17.1 nl/s, and that of the ejection stage is 5.6 ± 2.4 nl/s, as depicted in the inset of Fig. 3. In addition, the average flow rate of *An. sinensis* is 12.7 ± 1.7 nl/s and 0.7 ± 0.7 nl/s in the intake and ejection stages, respectively. The average flow rates of *Ae. togoi* at the intake and ejection stages are much higher than those of *An. sinensis* (P < 0.05), whereas the pumping frequencies of the two mosquitoes are comparable to a certain degree. The stroke volumes of *Ae. togoi* and *An. sinensis* are comparable because the higher intake and ejection volumes of *Ae. togoi* induce the zero-sum effects. However, the ratio of the ejection volume in *An. sinensis* is lower than that in *Ae. togoi* (P < 0.01).

### Wall shear stress of liquid-feeding flow

Many pathogens have used the adhesive structure of the vessel conduits of a host for their safety and reproduction^28^. Wall shear stress is a tangential force exerting between the flowing liquid and vessel wall. To allow the movement of the pathogens in the host’s vessel, this flow-induced tangential force should be higher than the threshold value of the adhesive force^29^,^30^. As such, the wall shear stresses (WSSs) in the proboscis of *Ae. togoi* and *An. sinensis* were evaluated to estimate the effects of their flow characteristics on pathogen transmission. The WSS can be expressed as a function of flow rate by using the following relationship of Hagen-Poiseuille flows:


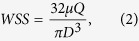


**Figure 4:**
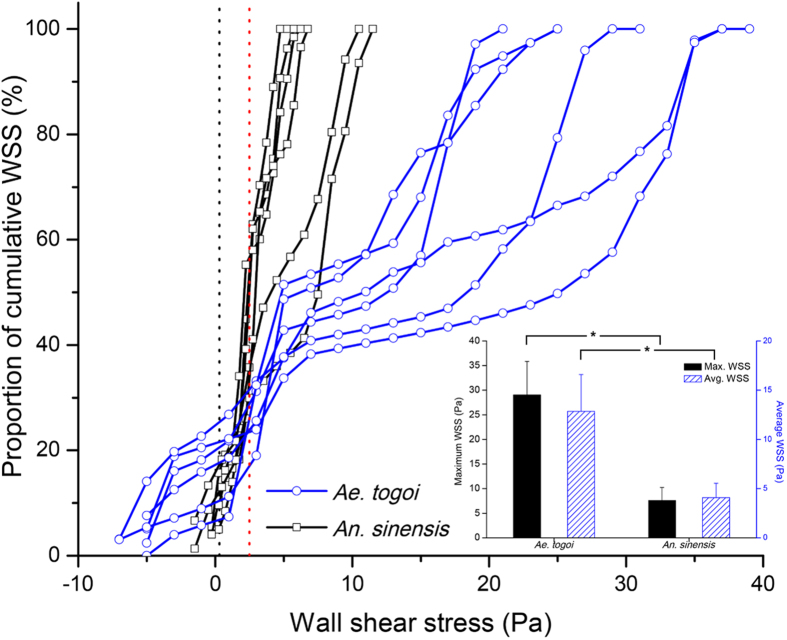
Variations of wall shear stress (WSS) between *Ae. togoi* and An. sinensis, as well as the corresponding cumulative proportion in one feeding cycle. The maximum WSS and average WSS are plotted in inset. A black dotted line indicates the WSS of 0.3 Pa, which is the physiological value required to intake iRBCs through a venule^30^,^31^. A red dotted line denotes the WSS of 2.5 Pa required to transport iRBCs adhered to a vessel^30^. Error bars represents the standard deviation; *P < 0.01.

Figure 4 shows the WSS distributions in *Ae. togoi* and *An. sinensis* and their cumulative proportions in one feeding cycle. To compare their pumping performance associated with the detachment of pathogens adhered to the host vessel, the WSSs of the two mosquito species were compared with the response of malaria-infected red blood cells (iRBCs) on WSS^30^,^31^. The transport of iRBCs through a vessel can endure up to the WSS of 0.3 Pa, a physiological condition of venules^30^,^31^. Once adhered, iRBCs remain stationary up to a WSS of 2.5 Pa^30^. The inset in Fig. 4 compares the maximum WSS and the average WSS of *Ae. togoi* and *An. sinensis* in one feeding cycle. The maximum WSSs of *Ae. togoi* and *An. sinensis* are 29.0 ± 6.8 Pa and 7.6 ± 2.7 Pa, respectively (P < 0.01). The average WSS of *Ae. togoi* (12.8 ± 3.7 Pa) is higher than that of *An. sinensis* (4.1 ± 1.5 Pa) (P < 0.01). The proportions of WSS over 0.3 Pa during feeding are 65.6–84.7% and 74.7–89.6% for *Ae. togoi* and *An. sinensis*, respectively. Moreover, the proportion of WSS over 2.5 Pa for *Ae. togoi* (68.5–82.9%) is much higher than that of *An. sinensis* (4.2–46.2%).

The dynamic motions of RBCs around the proboscis are closely related with the liquid-feeding ability of two mosquito species. Thus, the planar flow rates of RBCs were measured by integrating the flow velocity along the circular boundary at 80 μm away from the tip of the proboscis for comparison (Fig. S1 a–d)^32^. The maximum planar flow rate of RBCs in *Ae. togoi* (0.837 ± 0.052 mm^2^/s) is higher than that in *An. sinensis* (0.233 ± 0.008 mm^2^/s) (P < 0.01). Table 1 compares the liquid-feeding flow characteristics of four mosquito species, namely, *Ae. togoi, An. sinensis, Ae. aegypti*^33^*, and Cx. pipens pallens*^34^. Their liquid-feeding parameters, including proboscis diameter, average flow rate, and average WSS, vary distinctly.

### Morphological features of the pump systems

**Figure 5:**
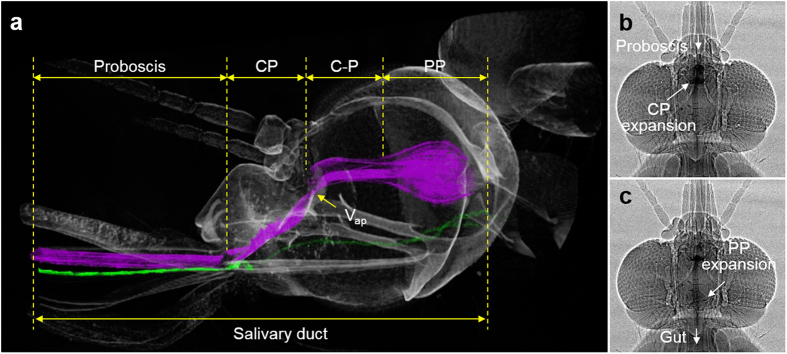
Typical morphology of the heads of a female mosquito (**a**), and sequential expansion and contraction of the CP (**b**) and PP (**c**) pumps. Figures show the functional features of the pump systems of *An. sinensis.* 3D-reconstruction image of the head part and sequential images of the pumps were obtained using X-ray micro-imaging technique.

To understand the different liquid-feeding flow characteristics of *Ae. togoi* and *An. sinensis*, the 3D structural features of the pump systems of the two mosquito species were observed by SR-μCT. The 3D reconstruction images of the head part of the two species were used to compare the morphological structures of the two pump organs. As shown in Fig. 5a, a long proboscis is serially connected to the cibarial pump (CP) and the pharyngeal pump (PP). The anterior pharyngeal valve (V_ap_) is located in front of the conduit connecting the two pump organs (C-P) to minimize the reversal flow toward the CP chamber^15^. The salivary duct is positioned under the liquid-feeding pump system. The structural parameters (*D*, *L*) used in the Hagen-Poiseuille relationship were obtained from the 3D reconstructed images of four similarly sized mosquitoes. The proboscis diameter of *Ae. togoi* (28.5 ± 3.1 μm) is smaller than that of *An. sinensis* (36.8 ± 4.2 μm) (P < 0.05). The other lengths of the two mosquito species are comparable (Table 2) (P > 0.05).

### Dynamic behaviors of pump systems

**Figure 6:**
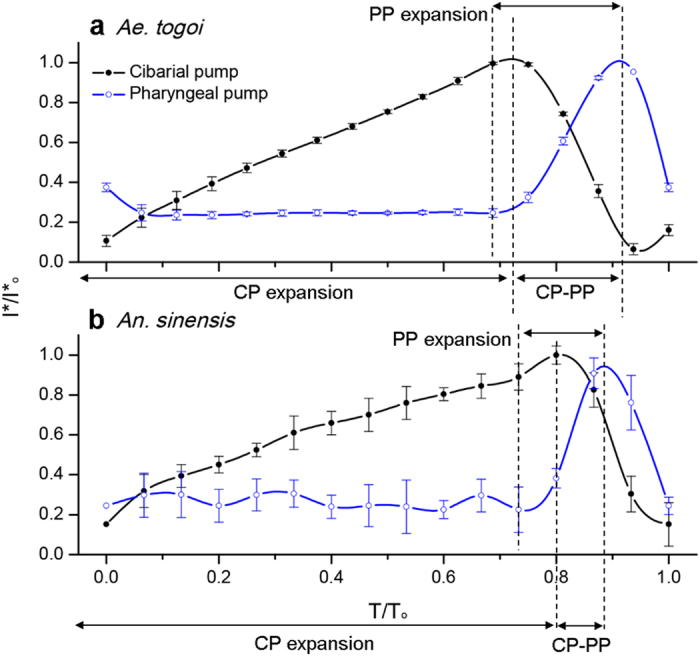
Systaltic volume variations of the pumping organs of *Ae. togoi* and *An. sinensis*. Volume variations in the two pumping organs of *Ae. togoi* (**a**) and *An. sinensis* (**b**). T/T_o_ represents the dimensionless time normalized by the liquid-feeding period T_o_. I^*^/I^*^_o_ represents the normalized light intensity. Error bars represents the standard deviation.

A large suction pressure (Δ*P*) is generated by the systaltic movements of the dilator muscles of the two pump organs^14^,^18^. Figures 5b,c show the typical systaltic motions of the two pump organs visualized by X-ray micro-imaging. As depicted in Fig. 6a,b, CP and PP are operated with a certain phase shift to regulate liquid-feeding^19^. The early contraction of the CP matches well with the abrupt decrease in pressure ratio estimated from the velocity information measured by micro-PIV (Fig. S2a,b). The volume of the CP is gradually increased during intake. Meanwhile, the PP expands near the end of the CP expansion. The flow moving toward the mosquito’s gut is regulated at the ejection stage^19^. As shown in Fig. 6a,b, the proportion of the CP expansion of the two mosquito species are comparable (76.2 ± 7.6% and 79.7 ± 3.8% for *Ae. togoi* and *An. sinensis*, respectively, P > 0.05). The phase shifts between the CP and PP (peak-to-peak delay) are also comparable (17.2 ± 11.1% and 14.3 ± 6.7% for *Ae. togoi* and *An. sinensis*, P > 0.05). In addition, the proportion of the PP expansion in *Ae. togoi* (21.2 ± 3.7%) is larger than that of *An. sinensis* (13.2 ± 5.8%) (P < 0.1).

## Discussion

The liquid-feeding phenomenon of female mosquitoes can help understand the transmission of pathogens. The flow characteristics of the liquid-feeding phenomenon can be determined by examining the parameters of the feeding liquids, flow in the proboscis, morphology, and dynamic behavior of the pump organs of mosquito species^14^. *Ae. togoi* and *An. sinensis* are separate subfamilies of mosquitoes, which exhibit different behavior^35^,^36^ and spread different types of pathogens^36^. The differences in food handling may provide distinctly different pathogen transmission. Thus, the liquid-feeding abilities of *Ae. togoi* and *An. sinensis* were comparatively investigated to understand the relationship between the liquid-feeding flow characteristics and pathogen transmission.

The intrinsic liquid-feeding flow characteristics of mosquitoes may affect the vector efficiency^7^. The pumping systems of mosquitoes directly or indirectly manipulate the transmission of pathogens. The infection rate and transmission efficiency of pathogens, which are main indexes of the vector efficiency^4^,^5^, are also influenced by the mosquito-induced flow. Therefore, pathogens can potentially manipulate the liquid-feeding abilities of mosquitoes for their successful transmission^7^,^9^,^10^,^11^,^12^,^13^. Consequently, the different liquid-feeding abilities of female mosquitoes can be used to determine the vector efficiency.

The liquid-feeding flow characteristics of *Ae. togoi* and *An. sinensis* were evaluated by micro-PIV. Micro-PIV has frequently been employed to investigate the liquid-feeding dynamics of mosquitoes^18^,^21^. Although it is difficult for conventional methods to measure the temporal variation of the liquid-feeding process in a feeding cycle^4^, this micro-PIV can provide instantaneous velocity field information of the flow related with the pathogen/host dynamics during the liquid-feeding process.

The two mosquito species tested in this study exhibit distinct differences in the range of flow rates and the ratio of ejection volume in one feeding period. The stroke volume and the ejection volume ratio were evaluated from the measured flow rates of the two mosquito species. The liquid-feeding pumps and salivary duct are located close to each other. The two pump systems are operated systaltically during liquid-feeding^14^. In addition, the pathogens re-ingested by the liquid-feeding mosquitoes play a key role in pathogen transmission^17^,^18^. Therefore, the proper manipulation of the stroke volume per feeding cycle and the ejection volume ratio are essential for pathogens to enhance the success of transmission^7^,^8^,^16^. The stroke volumes of *Ae. togoi* and *An. sinensis* are comparable, suggesting that their durations of salivation are similar during liquid-feeding. However, the ejection volume ratios of the two species are statistically different. This difference affect re-ingestion because the re-ingested pathogens can escape from the mosquito to a host during the ejection stage. Therefore, the interactions of two liquid-feeding flow characteristics can potentially influence the transmission efficiency of pathogens.

Pathogens manipulate the liquid-feeding behaviors of mosquitoes^7^. However, they also build up adhesive structures on the vessel conduits of a host for their safety and reproduction^28^. The infection rate of mosquito species can be strongly influenced by liquid-feeding pumping abilities to detach the sick cells adhered to the host vessels. Threshold shear stress is required to detach the sick cells^24^; thus, the WSS is among the main determinants of infection rate. The WSSs induced by *Ae. togoi* and *An. sinensis* were also compared with the adhesive forces of the iRBCs^30^,^31^. Both mosquito species are not in trouble to detach i-RBCs adhered to the wall surface of the host. However, the detaching ability of *Ae. togoi* exhibits superiority to that of *An. sinensis* because *Ae. togoi* presents a smaller diameter (*D*) and higher flow rate (*Q*) than those of *An. sinensis* (Eq. (2)). This expectation agrees with the results for the average numbers of sucked Brugia malayi microfilariae of each infected mosquito (13.06 and 4.23 for *Ae. togoi* and *An. sinensis*, respectively)^27^. In addition, the infection rate of *Ae. togoi* on the Brugia malayi microfilariae is greater than that of *An. sinensis* (90% and 65% for *Ae. togoi* and *An. sinensis*, respectively)^27^. Many mosquito species apparently demonstrate different liquid-feeding characteristics, as summarized in Table 1 for four different species. The liquid-feeding flow characteristics can potentially influence their infection rate. For example, *Cx. pipiens pallens* (87.5%) presents a higher infection rate of *Wuchereria bancrofti* than *Ae. togoi* (20%)^37^. In addition, *Ae. togoi* yields a higher infection rate of *chikungunya* than *Ae. aegypti*^38^.

To understand the different liquid-feeding flow characteristics of *Ae. togoi* and *An. sinensis*, the functional features of their pump systems were investigated by X-ray micro-imaging. The two mosquitoes belong to different subfamilies with different life activities; however, many functional features of the pump systems are comparable to a certain degree. Moreover, *Ae. togoi* and *An. sinensis* exhibit distinct liquid-feeding flow characteristics. A similar liquid-feeding tendency is observed in Prolixus species, the vector of *Chagas disease*^22^.

The temporal variations in the pump volume and proboscis diameter can be used to represent the different liquid-feeding flow characteristics. Owing to similar systaltic movements of the two pump organs of *Ae. togoi* and *An. sinensis*, the flow characteristics of the two mosquito species are similar. However, the net volume changes in the pumps of *Ae. togoi* are larger than those of *An. sinensis*^39^. The systaltic motion of pumping muscles generates the suction pressure (Δ*P*)^14^; thus, *Ae. togoi* can exhibit intake at increased flow rates in the intake stage. In addition, the larger volume shrinkage of *Ae. togoi* leads to an increased flow rate in the ejection stage. The proboscis diameter is closely associated with the flow rate and WSS in the proboscis, in consideration of the Hagen-Poiseuille relationship.

The movement and adhesion of pathogens are important for successful reproduction. Because pathogens live in environments dominated by viscosity effect, their own motion and the binding force of their adhesive structure would be not so significant^40^. Thus, the feeding phenomena of external liquid food of mosquitoes would mainly influence on the movement and adhesion of pathogens. *Ae. togoi* generates higher sucking pressure with larger net volume variation of pump organs, compared to *An. sinensis*^39^. This implies that *Ae. togoi* might easily facilitate the movement of pathogens with the help of higher intake flow rate. In addition, *Ae. togoi* easily detaches pathogens by applying higher tangential force (WSS) over the binding force of actin-based adhesion structures of pathogen^41^. Using this higher WSS, *Ae. togoi* would suck larger number of pathogens, compared to *An. sinensis*. These fluid dynamic aspects are associated with the infection rate of microfilariae (*Ae. togoi*: 90%, *An. sinensis*: 65%)^27^.

In conclusion, the liquid-feeding flow phenomenon in female mosquitoes is the key to understand pathogen transmission. Trade-offs occur between vector mosquitoes and pathogens, and they are closely associated with the liquid-feeding flow phenomenon, reproduction, and pathogen transmission. However, the effects of the liquid-feeding flow characteristics on pathogen transmission are rarely reported yet. In the current study, the liquid-feeding flow characteristics of *Ae. togoi* and *An. sinensis* were compared by SR-μCT and micro-PIV. The two mosquito species show distinctly different liquid-feeding characteristics but exhibit similar functional features. The flow characteristics, particularly WSS, influence the vector efficiency of female mosquito species. This study on pathogen transmission from fluid-mechanical perspective can facilitate understanding of vector-pathogen interactions and the overall infection process of vector-borne diseases.

## Methods

### Preparation of mosquito samples

Mosquitoes and larvae (*Anopheles sinensis s.l*. and *Aedes togoi*, Theobald 1907) were reared in an air-conditioned environment at 27 °C and 80% RH, with a 16/8 h day/night cycle. Larvae were fed with a slurry of ground fish food and transferred to a net cage prior to emergence. Mosquitoes were fed with 10% sucrose solution. Female mosquitoes at 3 days post-emergence were starved for 12 h prior to the experiment. Wing lengths of the tested female mosquitoes were measured microscopically as their representative indices body size^42^. The opaque labium of each female mosquito was removed by microsurgery to visualize the flow inside the food canal of the proboscis^18^,^21^.

### Flow measurements

Velocity fields of the flow passing through the food canal were measured by micro-PIV. Because the non-Newtonian property of blood has little influence on the liquid-feeding dynamics of the two mosquito species^14^, sucrose solution with 1% (w/w) concentration was used as working fluid. The sucrose solution seeded with fluorescent tracer particles of 1.0 μm in diameter (Molecular Probes, Eugene, OR, USA) to extract velocity field information with high accuracy. Viscosity of the sucrose solution measured using a digital viscometer (Brookfield DV − II + Pro, Brookfield Engineering Laboratory Instruments, Middleboro, MA, USA) was 1.5 cP at 25 °C. The food canal was immersed into a pool containing the sucrose solution. The food canal was illuminated with a continuous Nd:YAG laser (λ = 532 nm, SLOC, Shanghai, China) during liquid-feeding. The fluorescent image (λ = 554 nm) of tracer particles passed through an objective lens (M = 20, N.A. = 0.5) after filtering with an optical filter (λ = 550 nm) attached on a microscope (Eclipse 80i, Nikon, Tokyo, Japan). Flow images of migrating particles were captured using a high-speed charge-coupled device camera (Photron Ultima APX, Fujimi, Tokyo, Japan) at 10000 frame/s with a spatial resolution of 256 × 128 pixels. To confirm the traceability of the tracer particles in the flows, the Stokes number *St* (*St* = *ρ*_*p*_*a*^2^ *U*_*mean*_*/18μD*) was estimated, where *ρ*_*p*_ is the density of the tracer particles, *a* is the particle diameter, *μ* is the viscosity of the working fluid, *U*_*mean*_ is the mean velocity of the flow and *D* is the internal diameter of the food canal^43^. The Stokes number was approximately 1.3 × 10^−3^, indicating that the tracer particles conform to the flows. All experiments were conducted in an air-conditioned environment maintained at 25 °C and 45% RH. To enhance the signal-to-noise ratio, the average light intensity of the sequential images was subtracted from the instantaneous intensity of the sequential images. A fast Fourier transform based cross-correlation PIV algorithm was applied to two consecutive flow images to obtain the corresponding vector field of the liquid-feeding flow. Accurate velocity fields information can be obtained by adopting a multiple pass-processing procedure.

### Data processing

The average of the velocity vectors measured in the central plane of the proboscis was determined and then used to estimate the flow rate in the proboscis under the assumption of the Hagen-Poiseuille flow. The measured instantaneous flow rates were low-pass filtered with a cut-off frequency of 100 Hz^20^. The flow rates were phase-averaged to compare the liquid-feeding flow characteristics of *Ae. togoi* and *An. sinensis*. Polynomial interpolation was adopted to provide the same number of data points for each feeding cycle. The phase-averaged flow rates of six mosquito samples of the same body size were statistically analyzed. The WSSs were also evaluated under the assumption of the Hagen-Poiseuille flow^14^,^44^.

### Movement of two pump organs

Temporal variations in the morphological structures of the two pump organs inside the head of mosquitoes were observed by X-ray microscopy at the beamline 6C Bio Medical Imaging of the Pohang Light Source-II (Pohang, Korea). To examine the liquid-feeding process, the heads of the mosquitoes were exposed to a monochromatic X-ray beam with a peak energy of 24 keV. By feeding the mosquitoes with a diluted iodine solution^19^, X-ray images were consecutively recorded using an image detector (AndorZyla) with a spatial resolution of 2560 × 2160 pixels. The detector was equipped with a 30 μm-thick YAG:Ce scintillation crystal. Information regarding the phasic volume variation of the two pump chambers was obtained from the temporal variations of light-intensity in the captured X-ray images^45^. In addition, to evaluate the structural factors of the mosquito pumps, 2D slice images of the two mosquitoes were obtained by rotating a sample stage from 0° to 180° with intervals of 0.5°^46^. Octopus Imaging Software was used to obtain 3D-reconstruction images of the mosquito heads.

### Statistical analysis

All results are represented as the mean ± s. d. To compare the differences between *Ae. togoi* and *An. sinensis*, all data were statistically analyzed by using One-way analysis of variances (One-way ANOVA) for independent samples (*k* = 2).

## Additional Information

**How to cite this article**: Lee, S. J. *et al.* Peculiar liquid-feeding and pathogen transmission behavior of *Aedes togoi* and comparison with *Anopheles sinensis*. *Sci. Rep.* **6**, 20464; doi: 10.1038/srep20464 (2016).

## Supplementary Material

Supplementary Information

## Figures and Tables

**Table 1:** Different flow characteristics of four mosquito species.

Species	Diameter (μm)	Average flow rate (nl/s)	Average WSS (Pa)
*Ae. togoi*	28.5 ± 3.1	21.3 ± 12.2	12.9 ± 3.7
*An. sinensis*	36.8 ± 4.2	11.8 ± 1.8	4.1 ± 1.5
*Ae. aegypti*	30	7.1–14.2	4.0–8.1
*Cx. pipiens pallens*	21	12.6	20.8

Data were collected from *Ae. aegypti*^33^ and *Cx. Pipiens pallens*^34^. Data on *Ae. togoi* and *An. sinensis* are expressed as mean ± s. d.

**Table 2:** Comparison of the structural parameters between *A. sinensis* and *A. togoi*.

Species	Prob. length (μm)	CP length (μm)	C-P length (μm)	PP length (μm)
*An. sinensis*	2,323.4 ± 236.4	444.4 ± 70.2	174.9 ± 28.7	389.2 ± 59.8
*Ae. togoi*	2,168.3 ± 126.8	445.5 ± 172.2	234.7 ± 59.0	411.5 ± 119.3

Prob. proboscis; CP, cibarial pump; C-P, conduit connecting the cibarial and pharyngeal pumps; PP, pharyngeal pump; Diam, diameter. All data are expressed as mean ± s. d.
