# Supplementary material for: Peculiar liquid-feeding and pathogen transmission behavior of Aedes togoi and comparison with Anopheles sinensis
Source: Sci Rep. 2016 Feb 3;6:20464. doi: 10.1038/srep20464 (PMC4738329; doi:10.1038/srep20464)
Supplement: Supplementary Information [file srep20464-s1.pdf]

Scientific Reports

Supplementary information

Peculiar liquid-feeding and pathogen transmission behavior of *Aedes togoi* and comparison with *Anopheles sinensis*

Sang Joon Lee[1, 2, 3,*], Dooho Kang[1,2], Seung Chul Lee[2,3] & Young-Ran Ha[2,4]

[1]Department of Integrative Biosciences and Biotechnology, Pohang University of Science and Technology, Pohang, 790-784, Republic of Korea

[2]Center for Biofluid and Biomimic Research, Pohang University of Science and Technology, Pohang, 790-784, Republic of Korea

[3]Department of Mechanical Engineering, Pohang University of Science and Technology, Pohang, 790-784, Republic of Korea

[4]Division of Integrative Bioscience and Bioengineering, Pohang University of Science and Technology, Pohang, 790-784, Republic of Korea

[*]Corresponding author: **Prof. Sang Joon Lee**

Center for Biofluid and Biomimic Research, Department of Integrative Biosciences and Biotechnology, Department of Mechanical Engineering, Pohang University of Science and Technology, San 31, Hyojadong, Namgu, Pohang, Kyungbook, 790-784, Republic of Korea

Tel.: +82-54-279-2169, Fax: +82-54-279-3199, E-mail: sjlee@postech.ac.kr

Supplementary

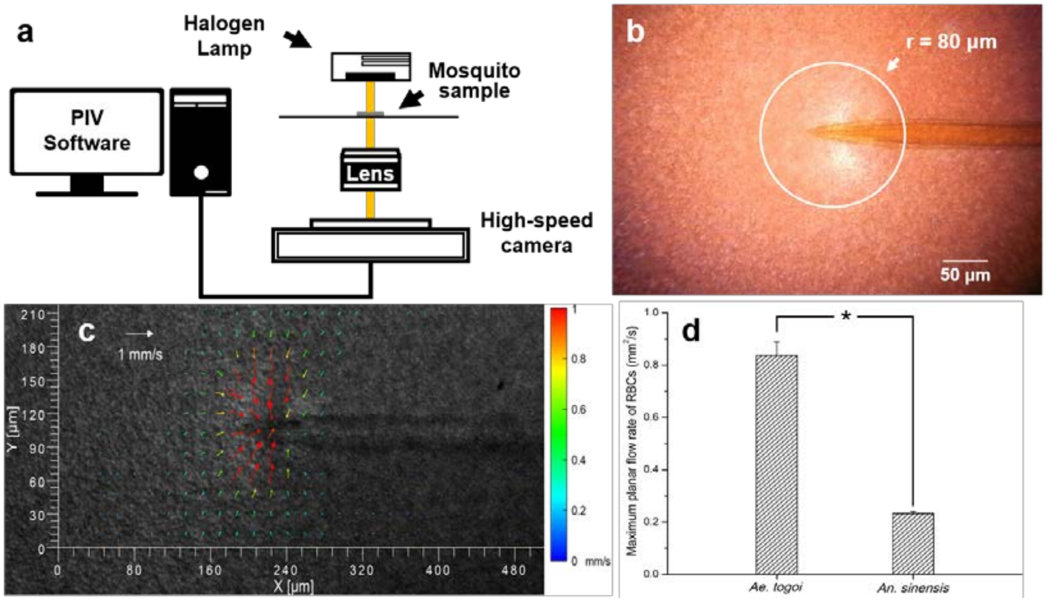

Figure S1. Comparison of the maximum planar flow rates of RBCs in *Ae. togoi* and *An. sinensis*.

The maximum planar flow rate of RBCs in *Ae. togoi* and *An. sinensis* were evaluated by measuring velocity information using micro-PIV. (a) A schematic diagram of the experimental set-up, (b) typical image of flow around the tip of proboscis, (c) velocity field around the proboscis at an the intake phase where the intake-flow rate is maximum, (d) comparison of the maximum planar flow rates of RBCs in *Ae. togoi* and *An. sinensis*. The velocity vectors inside the circle of figure (b) were integrated to evaluate the planar intake rate of RBCs. Error bars represents the standard deviation; *P < 0.01.

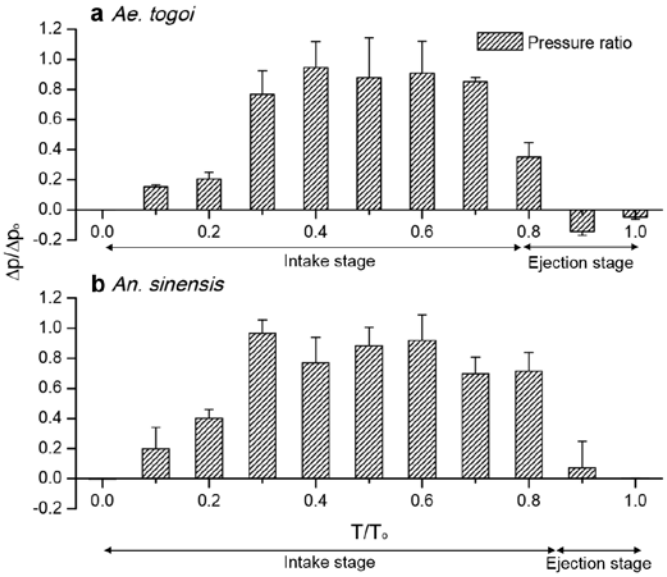

Figure S2. Variations of the pressure ratio of *Ae. togoi* (a) and *An. sinensis* (b).

The variations of the pressure ratio of *Ae. togoi* and *An. sinensis* were evaluated from the velocity information measured by micro-PIV technique. T/T_o represents the dimensionless time normalized the liquid-feeding period T_o. $\Delta P/\Delta P_o$ represents the pressure ratio normalized the maximum suction pressure ΔP_o. Error bars represents the standard deviation.
